# Differential Effects of Individually Adapted Challenge‐Oriented Heidelberg Ballschule Activities Versus Static Version on Motor Competence and Affective Engagement in 9–11‐Year‐Old Female Children

**DOI:** 10.1155/bmri/5244454

**Published:** 2026-05-26

**Authors:** Neda Mohammad Veysi, Ayoob Sabaghi, Behrooz Ebrahimi

**Affiliations:** ^1^ Department of Motor Behaviour, Faculty of Sport Sciences, Razi University, Kermanshah, Iran, razi.ac.ir

**Keywords:** affective engagement, challenge point framework, Heidelberg Ballschule, motor competence, physical activity, preadolescent girls

## Abstract

The widespread decline in motor competence and habitual physical activity (PA) among children underscores the need for innovative, intrinsically motivating pedagogical interventions. This quasiexperimental study examined the differential effects of two variants of the Heidelberg Ballschule program—one featuring individually adapted, challenge‐oriented progression aligned with participants′ optimal challenge points, and the other using static, nonprogressive tasks—on motor competence, affective engagement (enjoyment of PA), and weekly PA levels in 9–11‐year‐old female children in Kermanshah, Iran. Thirty girls aged 9–11 years were randomly assigned to either the challenge‐oriented group (*n* = 15) or the static group (*n* = 15). Both groups engaged in Heidelberg Ballschule ball activities for 8 weeks (three sessions/week, 45–60 min/session). Motor competence was assessed pre‐ and postintervention using a standardized tool (e.g., Bruininks–Oseretsky Test of Motor Proficiency), affective engagement via a validated enjoyment questionnaire, and PA levels throughout the intervention period via self‐report (Physical Activity Questionnaire for Children [PAQ‐C]). Results indicated that the challenge‐oriented group significantly outperformed the static group in overall motor competence (including gross and fine motor components), reported higher levels of affective engagement/enjoyment during PA, and exhibited greater weekly PA levels. These findings provide preliminary evidence that individually adapted, challenge‐oriented Heidelberg Ballschule activities—grounded in the challenge point framework—effectively enhance motor competence, foster positive affective responses to PA, and promote increased PA participation in preadolescent girls. Such approaches hold promise for integration into school‐based physical education programs to address motor and activity deficits.

## 1. Introduction

The health of contemporary children is profoundly shaped by environmental shifts and lifestyle transformations [1]. Factors such as urban expansion, diminished access to play spaces, and the predominance of sedentary digital entertainment have given rise to two interconnected challenges: rising rates of childhood obesity and widespread deficiencies in fundamental motor competence [1, 2]. Research indicates these deficiencies manifest particularly as impairments in coordination, balance, and object control [3]. The displacement of dynamic, communal play by solitary, inactive pursuits, compounded by a lack of engaging and effective movement opportunities in schools, has not only reduced children′s daily physical activity (PA) levels but also disrupted the natural acquisition of motor competencies [4, 5]. Contrary to common belief, motor competence is not an automatic byproduct of biological maturation; rather, it requires the deliberate design of learning environments, the provision of targeted practice aligned with the child′s developmental level, and appropriate instructional guidance [6, 7]. Motor competence encompasses gross motor skills (e.g., locomotion and throwing) and fine motor skills (e.g., precise manipulation and visuomotor integration), both of which form the basis for daily functioning, sport involvement, and long‐term physical literacy [8, 9]. Consistent engagement in PA bolsters underlying attributes such as balance, strength, coordination, and cardiorespiratory capacity [10–12]. Yet, recent evidence emphasizes that the quality and pedagogical design of practice are as critical as activity volume for promoting durable motor learning, affective engagement, and sustained participation [13, 14]. Conventional, teacher‐directed physical education approaches, grounded in direct instruction and repetitive drills, often achieve short‐term technical accuracy but fall short in cultivating intrinsic motivation, enjoyment, retention, and real‐life transfer [15, 16]. In contrast, game‐based learning (GBL) embodies constructivist principles by situating skill development within engaging, interactive, and playful contexts that encourage autonomy, decision‐making, creativity, and social collaboration [17, 18]. Systematic reviews and meta‐analyses indicate that GBL yields moderate‐to‐large benefits for motor and cognitive outcomes while markedly enhancing enjoyment, self‐efficacy, and voluntary involvement—effects especially evident in children and briefer interventions [19, 20]. Consequently, game‐based formats are increasingly viewed as superior when objectives encompass holistic development and lifelong adherence beyond isolated performance gains [21, 22]. Research on challenge‐based versus nonchallenge interventions for motor competence remains sparse and predominantly limited to isolated, sport‐specific drills delivered through direct instruction [23, 24]. The present study bridges this gap by integrating the challenge point framework (CPF) [25]—which posits optimal learning when task difficulty is dynamically calibrated to the individual′s skill level—within a comprehensive, playful Heidelberg Ballschule curriculum. This combination of GBL with individualized challenge adaptation represents a novel empirical synthesis. Structured ball activities provide a multifaceted developmental medium, simultaneously advancing motor attributes (balance, agility, and coordination) and cognitive processes (tactical awareness and problem‐solving) while naturally supporting socioemotional growth through interaction [26, 27]. Their adaptable, enjoyable format is well‐suited to fostering intrinsic motivation and positive attitudes toward active lifestyles [28, 29]. The Heidelberg Ballschule program, developed in Germany by Klaus Roth, exemplifies this approach through diverse, non‐sport‐specific minigames using varied balls to build a foundational “motor alphabet” via exploration, variability, and progressive challenge [30]. Empirical work demonstrates its advantages over traditional methods in motivation, enjoyment, and broad motor competence [31], rendering it particularly appropriate for early interventions targeting fundamental movement literacy [32]. Affective engagement in PA—encompassing positive emotional experiences such as pleasure, fun, and satisfaction—arises when environments align with innate needs for autonomy, mastery, and relatedness [33, 34]. Programs incorporating choice and appropriately scaled challenges consistently yield higher enjoyment, intrinsic motivation, and adherence in children [34, 35]. Given the pivotal role of motor competence in child development and the promise of structured ball games for its enhancement, this quasiexperimental study investigates the differential effects of an 8‐week Heidelberg Ballschule intervention delivered in an individually adapted, challenge‐oriented condition versus a static, nonprogressive version on motor competence, affective engagement (enjoyment of PA), and weekly PA levels among 9–11‐year‐old female children in Kermanshah, Iran. By manipulating task challenge within a game‐based framework, the research offers new insights into optimizing both motor learning efficacy and affective responses in elementary physical education, with implications for promoting sustained PA and countering sedentary trends.

## 2. Methods

### 2.1. Study Design

The present study employed a quasiexperimental design with a pretest and posttest structure. This design was selected to assess the comparative effects of the two intervention strategies on the dependent variables.

### 2.2. Population, Sample, and Ethical Considerations

This quasiexperimental investigation focused on a population of 9–11‐year‐old female students enrolled in public elementary schools across Kermanshah, Iran, in the 2023–2024 school year. An a priori power analysis was conducted to determine the minimum required sample size, assuming a large effect size (Cohen′s *f* = 0.54) based on prior motor intervention studies in children [36], an alpha level of 0.05, and a desired statistical power of 0.80. For a one‐way analysis of covariance (ANCOVA) with two independent groups and three covariates (pretest score, age, and body mass index [BMI]), the analysis indicated that a total sample size of 26 participants would be sufficient to detect the specified effect. To account for potential attrition and to enhance parameter stability—particularly given the pediatric population and motor skill assessment context—the final sample was increased to 30 participants (15 per group), yielding an actual power of approximately 0.85. This sample size aligns with previous intervention studies in this field [23, 31]. The study protocol received full ethical approval from the Institutional Review Board of Razi University (Ethics Code: IR.RAZI.REC.1402.039). Prior to enrollment, written informed consent was obtained from parents or legal guardians, and developmentally appropriate verbal assent was secured from all child participants following a simplified oral explanation of the study aims, procedures, and voluntary nature of participation. Participants and their guardians were explicitly informed that withdrawal from the study at any stage would be permitted without consequence to their academic or extracurricular standing.

### 2.3. Data Collection Instruments

#### 2.3.1. Anthropometric Measurements

BMI was included as a covariate in the statistical analysis. BMI was derived using the standard formula: Weight (kg)/Hieght (m)^2^.

#### 2.3.2. Physical Activity Questionnaire for Older Children (PAQ‐C)

Weekly PA levels were monitored using the validated Persian adaptation of the PAQ‐C. This self‐report tool estimates habitual moderate‐to‐vigorous physical activity (MVPA) via a 7‐day recall format. The questionnaire comprises 10 items; the first nine items probe activity frequency across various domains (e.g., physical education, recess, and after‐school), while the tenth screens for illness‐related inactivity. Responses are recorded on a 5‐point Likert scale (1 = *low activity*, 5 = *high activity*), with a composite score derived from the mean of Items 1–9. The psychometric adequacy of this version for the target age group has been established [37].

#### 2.3.3. Bruininks–Oseretsky Test of Motor Proficiency (BOTMP)

The BOTMP is a widely utilized, standardized instrument designed to evaluate the motor performance of children and adolescents aged 4.5–14.5 years. For the purpose of this study, the Bruininks–Oseretsky Test of Motor Proficiency‐Short Form (BOT‐2 SF) was specifically utilized. This form is comprised of 14 items carefully selected from the complete version of the test to serve as an efficient screening tool. It provides a quick yet reliable assessment, yielding a general composite score that reflects overall proficiency across both gross and fine motor skills. Crucially, the administration of the BOT‐2 SF requires approximately 15–20 min per child and was conducted individually for each participant. The BOT‐2 SF is a standardized norm‐referenced scale that demonstrates adequate validity and high reliability, with the reliability coefficient for this specific form reported at 86% [38, 39].

#### 2.3.4. Assessment of Enjoyment of PA

**Figure 1:**
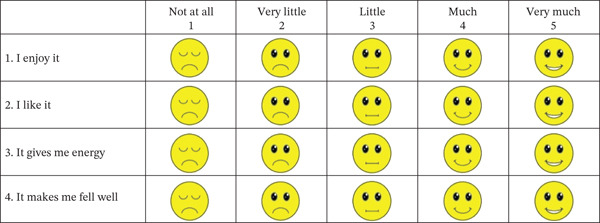
Scale used for assessing the enjoyment of PA.

Enjoyment derived from the PAs was measured via a brief, adapted instrument (Figure 1). Four items were selected from the longer PA Enjoyment Scale and modified for a pediatric sample by incorporating emoji‐based pictorial anchors to facilitate comprehension and reliable responding [34]. Participants rated their agreement with each statement on a 5‐point scale (1 = *not at all*, 5 = *very much*). The validity of this adapted scale for assessing children′s affective responses to PA is supported by existing literature [34, 40]. The scale has also demonstrated acceptable reliability, with a reported Cronbach′s alpha of 0.78 for the enjoyment questionnaire [40].

### 2.4. Procedure

The intervention lasted 8 consecutive weeks, comprising three standardized 60‐min sessions per week for each group, resulting in a total of 24 sessions. All sessions were delivered by a single qualified Ballschule instructor to maintain treatment fidelity, with consistent warm‐up protocols, equipment provision, and overall session duration across both conditions. The content for both groups was drawn from the same set of 15 foundational Heidelberg Ballschule games (Kröger & Roth, 2011), ensuring equivalence in core activities while differing solely in the application of task difficulty manipulation based on the CPF [25].

### 2.5. Static (Nonchallenge) Group

In the static (nonchallenge) condition (*n* = 15), all Heidelberg Ballschule games were conducted with fixed rules and constant task constraints throughout the full 8‐week intervention. No modifications, progressions, or individual adaptations were introduced at any stage; each participant consistently performed the exact same version of every game from the initial session to the final one. Representative examples of these unchanging tasks included balancing a ball on various body parts such as the elbow, outstretched hand, head, foot, or palm; throwing and catching with a partner from 3–4 m after one bounce, followed by performing a simple turn; throwing the ball vertically upward and maintaining it in the air solely with the back of the hand; and standing opposite a partner, simultaneously throwing balls vertically, switching positions, and catching each other′s ball.

### 2.6. Individually Adapted Challenge‐Oriented Group

In the individually adapted challenge‐oriented condition (*n* = 15), task difficulty was continuously personalized and adjusted in real time for each child during every session, with the clear objective of maintaining an optimal challenge point in accordance with the CPF. Participants regularly changed partners (re‐paired every 3–5 min in pairs or small groups) to increase variability and promote social interaction; however, all difficulty adjustments were made strictly on an individual basis, independent of the pair or group performance. The instructor followed a structured, performance‐driven adaptive protocol, monitoring each child continuously and recording observations on a simple clipboard tally sheet (one row per child per session). Adjustments were applied within 10–15 s based on immediate performance:

‐ When a child achieved ≥ 80% success across six to eight consecutive trials at the current difficulty level, the task was immediately made more challenging for that child only. Examples of progressions included shifting from basic hand balancing to balancing on the knee, arm, or between the shoulders; stacking and balancing two balls; performing the task while sitting down, standing up, or spinning; or incorporating additional motor or cognitive demands (e.g., showing and verbally announcing a number with fingers before catching, executing a 360° turn or jump–spin after throwing, using a smaller ball, catching while seated or jumping, or adding an active defender).
‐ When success dropped to ≤ 50% across six to eight consecutive trials, difficulty was immediately reduced for that individual. Examples of regressions included returning to a single larger ball, allowing two bounces, removing cognitive requirements, increasing the playing area, or eliminating the defender.
‐ Success rates between 51% and 79% resulted in maintaining the current individualized difficulty level until the next evaluation period.

This individualized progression system was consistently applied across all 15 games. Specific examples of the actual challenge progressions implemented are provided in the Supporting Information section.

### 2.7. Assessment Timeline

Preintervention measures—including anthropometric data, motor competence evaluation via the BOT‐2 SF, baseline PAQ‐C, and initial affective engagement/enjoyment ratings following a familiarization session—were collected 5–7 days prior to the start of the intervention. Weekly PA levels were assessed using the PAQ‐C at the conclusion of the final session each week (Weeks 1–8). Postintervention assessments (identical to the preintervention battery) were conducted 24–48 h after the final session to minimize acute fatigue effects while capturing peak training adaptations. All assessments followed standardized protocols, with assessors blinded to group allocation where feasible, to ensure objectivity and reliability.

## 3. Statistical Analysis

To describe the research variables and the general characteristics of the participants, descriptive statistics (mean and standard error of the mean) were employed. For testing the hypotheses of this study, ANCOVA was used. To examine the intervention′s effect more precisely on these factors, the results of the pretests along with the variables of age, BMI, and PA level during the intervention period—each of which can potentially influence motor proficiency [31, 41–45]—were considered as covariates to ensure that observed changes were primarily attributable to the type of intervention. Prior to conducting any of the tests, the assumptions of the tests were verified using the Shapiro–Wilk and Levene tests. Data analysis was performed using SPSS Version 25, with a significance level set at (*p* < 0.05).

## 4. Results

### 4.1. Demographic Characteristics and PA Levels

The demographic characteristics of the participants and their activity levels during the intervention are presented in Table 1. Also, one participant from the nonchallenge group was excluded from the final analysis due to nonparticipation in the posttest assessment. Therefore, the analysis was conducted on the remaining 29 participants. Initial independent samples *t*‐tests and the Mann–Whitney *U* test confirmed that the challenge and nonchallenge groups were statistically comparable at baseline across all measured demographic variables. Specifically, no significant differences were found for age (*p* = 0.93), height (*t*_(27)_ = 1.37, *p* = 0.18), weight (*p* = 0.98), or BMI (*t*_(27)_ = 0.63, *p* = 0.15), confirming a successful randomization for these control variables. The PA level reported in Table 1 represents the mean weekly PA level calculated across the entire 8‐week intervention period. Analysis revealed a statistically significant difference between the groups in this mean PA level (*t*_(27)_ = 3.02, *p* = 0.005). The challenge group reported a significantly higher average PA level (3.06 ± 0.18) compared to the nonchallenge group (2.40 ± 0.11) throughout the 8‐week program.

**Table 1:** Demographic characteristics and mean 8‐week PA levels of participants (mean ± standard error of the mean).

Variable	Challenge group	Nonchallenge group	Levene′s test		Independent sample *t*‐test or Mann–Whitney *U* test		
			*F*	*p*	*t*	df	*p*
Age (years)	9.93 ± 0.19	10.01 ± 021	—	—	—	—	0.93
Height (m)	1.33 ± 0.01	1.34 ± 0.01	0.39	0.53	1.37	27	0.18
Weight (kg)	32.40 ± 1.66	32.14 ± 1.31	—	—	—	—	0.98
BMI (kg/m^2^)	18.33 ± 0.7	17.71 ± 0.69	0.20	0.65	0	27	0.15
PA level during the intervention period	3.06 ± 0.18	2.40 ± 0.11	3.49	0.07	3.02	27	0.005

### 4.2. Pretest Comparison of Outcome Variables

To evaluate baseline equivalence between the challenge and nonchallenge groups, independent samples *t*‐tests were conducted on all outcome variables at pretest. As presented in Table 2, no statistically significant differences were observed between the groups in gross motor skills (*t*_(18.92)_ = 0.44, *p* = 0.66), fine motor skills (*t*_(27)_ = 1.59, *p* = 0.12), overall motor proficiency (*t*_(16.87)_ = 1.06, *p* = 0.30), or enjoyment of PA (*t*_(27)_ = 0.91, *p* = 0.36). These results confirm that the groups were comparable at baseline, supporting the assumption of initial equivalence required for subsequent ANCOVA.

**Table 2:** Independent *t*‐test results comparing outcome variables at pretest.

Factors	*t*(independent *t*‐test)	df	*p*
Gross motor skills	0.44	18.92	0.66
Fine motor skills	1.59	27	0.12
Motor proficiency (overall)	1.06	16.87	0.30
Enjoyment of PA	0.91	27	0.36

### 4.3. Descriptive and Inferential Results for Outcome Variables

Table 3 provides the descriptive statistics for motor proficiency components and enjoyment of PA at pretest and posttest. Posttest descriptive scores consistently show that the challenge group achieved higher average scores across all motor and psychological variables compared to the nonchallenge group.

**Table 3:** Mean scores of motor proficiency and enjoyment of PA in the study groups (mean ± standard error of the mean).

Factors	Challenge group		Nonchallenge group	
	Before	After	Before	After
Gross motor skills	24.66 ± 0.61	34.46 ± 0.64	25.28 ± 1.28	29.5 ± 1.21
Fine motor skills	8.13 ± 044	12.86 ± 046	9.21 ± 0.51	11.85 ± 0.59
Motor proficiency	32.80 ± 0.57	47.33 ± 0.83	34.50 ± 1.48	41.35 ± 1.56
Enjoyment of PA	3.16 ± 0.13	3.93 ± 0.40	3.01 ± 0.08	3.19 ± 0.36

To examine the effect of the intervention type while controlling for initial differences in pretest scores, BMI, and mean PA levels, ANCOVA was performed. Preliminary analysis confirmed that all necessary statistical assumptions were met. The ANCOVA results (Table 4) showed a significant main effect of intervention type across all outcome variables, indicating greater improvements in the challenge‐based approach. For gross motor skills, a significant difference was observed (*F*_(1)_ = 15.11, *p* = 0.001, *η* ^2^ *p* = 0.41) favoring the challenge group. Fine motor skills also showed a significant effect (*F*_(1)_ = 5.38, *p* = 0.03, *η* ^2^ *p* = 0.20). Overall motor proficiency demonstrated a highly significant improvement (*F*_(1)_ = 16.40, *p* = 0.001, *η* ^2^ *p* = 0.43). Similarly, enjoyment of PA was significantly higher in the challenge group (*F*_(1)_ = 16.85, *p* = 0.001, *η* ^2^ *p* = 0.44), indicating the greatest increase in enjoyment.

**Table 4:** Results of ANCOVA for examining between‐group differences in motor proficiency and enjoyment of PA.

Factors	df	*F*	*p*	*η* ^2^ *p*	Adjusted mean difference (challenge–nonchallenge)	95% confidence interval (CI)
Gross motor skills	1	15.11	0.001	0.41	5.24	[3.53, 7.12]
Fine motor skills	1	5.38	0.030	0.20	2.06	[0.64, 3.52]
Motor proficiency	1	16.40	0.001	0.43	7.65	[4.88, 10.43]
Enjoyment of PA	1	16.85	0.001	0.44	0.66	[0.33, 0.99]

## 5. Discussion

This investigation empirically contrasted two instructional variants within the Heidelberg Ballschule curriculum: one featuring dynamic, individualized challenge progression guided by ongoing performance feedback, and the other relying on static, unchanging tasks. Primary outcomes encompassed motor proficiency, enjoyment of PA, and self‐reported weekly PA levels among 7–9‐year‐old preadolescent girls. PA levels were additionally monitored across the intervention period. Participants in the challenge–progressive condition exhibited markedly superior motor proficiency, elevated enjoyment during PA, and higher self‐reported weekly PA relative to the nonchallenge condition. Such advancements arm children with foundational motor competencies that facilitate enhanced functioning in school physical education, athletic pursuits, and routine activities. Structured play‐based ball games, especially under progressive challenge guidance, hold considerable promise for supporting comprehensive developmental trajectories in youth. The pronounced superiority of the challenge‐oriented protocol is entirely consistent with the foundational principles of the CPF [25]. CPF outlines an inverted‐U function linking task difficulty to learning efficacy, asserting that motor acquisition and motivational engagement peak when functional task difficulty is continually and adaptively matched to the performer′s current proficiency, thus preserving an optimal challenge point [46]. Here, individualized real‐time progression criteria secured this alignment. Sustaining each participant at her personalized optimal challenge point throughout sessions concurrently (a) supplied the greatest volume of constructive error‐derived feedback and cognitive involvement indispensable for advanced motor learning [47, 48] and (b) upheld perceived competence alongside intrinsic gratification, thereby bolstering persistent enjoyment and exertion [46, 48]. Accordingly, the amplified improvements in motor proficiency and enjoyment within the challenge group constitute mechanistically intertwined results from ongoing CPF‐aligned optimal challenge maintenance.

These observations align with prior investigations showing that challenge‐driven interventions substantially outperform nonchallenging counterparts in advancing motor proficiency elements [23, 24]. Such protocols compel more swift adaptation and skill refinement via variability and abrupt perturbations. The current data broaden those precedents by illustrating effective CPF operation within an integrated game‐embedded program, yielding combined gains in motor execution and affective responses. Tasks engineered for suitable challenge heighten cognitive investment [49, 50], encourage more profound task information elaboration [51, 52], and stimulate problem‐solving capacities [53, 54]. These processes jointly refine motor learning through improved congruence between task demands and performer ability [25, 55, 56]. Appropriate difficulty‐induced cognitive demands also fortify executive functions like attention regulation and working memory, fostering superior motor–cognitive performance [55, 57]. Moreover, constraints‐led and guided‐discovery elements permit children to test varied solutions, accommodate fluctuations, and solidify learning more robustly [58, 59]. Neurophysiological underpinnings additionally elucidate the edge of challenge‐based settings. Demanding conditions amplify prefrontal cortex involvement and perceptual–motor efficacy [60, 61]. Errors arising therein function as informative negative feedback, enabling error‐driven adaptation and movement calibration [62]. Challenge encounters further cultivate resilience, prompting multifaceted task analysis, strategy innovation, and enriched comprehension [63–65]. Motivational self‐talk commonly surfaces in arduous segments to facilitate progression through hurdles [66]. A pivotal outcome revealed that challenge‐based Heidelberg Ballschule games substantially boosted PA enjoyment over nonchallenge counterparts. Games with calibrated challenge elicit heightened pleasure, subsequently amplifying involvement, exertion, and continuity [66]. Enjoyment stands as a cardinal psychological determinant of intrinsic motivation and sustained exercise commitment [67, 68]. Emerging literature frames enjoyment as a potent mediator of PA patterns, whereby perceiving activities as invigorating, arousing, and pleasurable correlates with greater regularity and volume of participation [69, 70]. The notably elevated enjoyment in the challenge arm ties mechanistically to CPF: sustaining tasks within reachable yet demanding bounds yielded recurrent achievements amid controllable pressures and optimized perceived competence and flow‐resembling positive states [71, 72]. Such positive affect elevates prospects for lasting exercise patterns, firmer continuance intentions, and increased PA frequency [73–75]. Heightened enjoyment likewise bolsters self‐efficacy and PA‐aligned conduct [76, 77], empowering higher‐efficacy individuals to apply intensified effort amid demands [78]. Enjoyable episodes provoke dopamine liberation, aiding memory stabilization and acquisition [79]. Favorable affect additionally advances synaptic potentiation, refining sensory–motor fusion and proficiency gains [80, 81]—factors collectively underpinning observed motor enhancements. This bidirectional reinforcing loop (elevated motor proficiency‐amplified enjoyment) furnishes a compelling rationale for the observed rise in self‐reported PA during intervention in the challenge condition. Nonetheless, reliance on the self‐report PAQ‐C instrument leaves room for social desirability bias. Participants in the more gratifying challenge setting—benefiting from amplified fun, accomplishment, and affirmative coaching—might have modestly inflated reports of activity or enjoyment to conform to anticipated norms or mirror authentic positive experiences. Although the directional group disparity coheres fully with objective motor proficiency advances and enjoyment metrics, the specific extent of PA elevation merits cautious interpretation. These insights stress that nurturing enjoyment via nurturing contexts, skill cultivation, and affirmative encounters constitutes an indispensable tactic for advancing routine PA and wellness [68, 69]. Motor proficiency undergirds routine operations and health‐oriented involvement [82], entailing precise, regulated, and synchronized movement production. Longitudinal data affirm that motor competence gains forecast later PA elevations and wider health advantages [83]. Amid escalating motor delay occurrences in youth [84], prioritizing early skill augmentation offers an efficacious pathway to PA augmentation [73, 85]. Hence, motor proficiency advancement warrants central status in physical education curricula and youth athletic initiatives to counter diminishing PA patterns and escalating obesity [83]. This work stands apart by centering ball games as the chief conduit for motor skill instruction. Diverging from earlier emphases on conventional physical education paradigms, it spotlights pleasurable, interactive ball pursuits. Relative to rigid, directive methodologies, play‐oriented formats confer enhanced adaptability and accentuate collaboration, inventiveness, and adaptive resolution—qualities rendering them more encompassing and enticing across child populations [86–88]. Considering the prevalent paucity in play–exercise variety, developmentally attuned and interest‐aligned programs are vital. Pleasurable instructional paradigms can markedly propel motor maturation in primary‐aged children and satisfactorily fulfill motor requisites [89, 90]. Play transcends mere pedagogy to serve as a cornerstone of child ontogeny, bolstering physical, cognitive, social, and affective spheres [91, 92]. Ball games particularly cultivate visuomotor alignment, force governance, and temporal sequencing [93]. Within the Heidelberg Ballschule paradigm, children incrementally assimilate the “motor alphabet”—paralleling alphabetic acquisition for literacy—gaining core movement tenets via playful engagement [30]. Incorporated challenges are meticulously scaled for apt difficulty. By weaving motor and cognitive intricacies into gameplay, activities sharpen targeted proficiencies (e.g., eye–hand synchronization, reaction latency, and force modulation) while spurring perpetual adjustment to emergent and fluctuating scenarios. The framework upholds efficacious equilibrium between extant child capability and game exigency—averting exhaustion/frustration from undue intricacy while sidestepping apathy from undue ease. This equilibrated architecture augments drive, perpetuates involvement, and invigorates vigorous game participation.

## 6. Limitations

Several limitations of the present study warrant consideration. The reliance on convenience sampling and a modest sample size (*N* = 30) reduces statistical power and constrains the generalizability of the results to more heterogeneous or larger populations. The investigation was restricted to 9–11‐year‐old female children, precluding direct extrapolation of findings to boys, other age groups, or mixed‐gender samples. PA levels were evaluated exclusively via self‐report using the PAQ‐C, a method susceptible to recall inaccuracies and social desirability bias; future research would benefit from incorporating objective measures such as accelerometers or pedometers across extended monitoring periods. Additionally, the intervention spanned only 8 weeks, offering no insight into the durability of gains in motor competence or sustained changes in PA behavior; longitudinal assessments at 6–12 months postintervention are recommended to determine the longevity of the observed effects. Notwithstanding these constraints, the current results offer compelling preliminary support for the superiority of an individually adapted, challenge‐oriented Heidelberg Ballschule approach in promoting motor competence, affective engagement, and weekly PA among preadolescent girls.

## 7. Conclusion

This 8‐week quasiexperimental study demonstrates that an individually adapted, challenge‐oriented variant of the Heidelberg Ballschule program produces significantly greater enhancements in motor competence, affective engagement (enjoyment of PA), and self‐reported weekly PA levels in 9–11‐year‐old female children compared to a static, nonprogressive version of the same curriculum. These differential advantages align closely with the principles of the CPF [25], in which continuous, real‐time calibration of functional task difficulty to the child′s evolving performance level simultaneously maximizes cognitive–motor learning—through optimal error information and heightened executive involvement—and intrinsic motivation—through elevated perceived competence and flow‐like positive affect. Accordingly, challenge‐oriented Heidelberg Ballschule activities represent a highly efficacious, play‐centered instructional model capable of concurrently advancing fundamental motor competence and nurturing favorable affective responses to PA—two critical protective elements against childhood physical inactivity and obesity. The findings provide a strong rationale for incorporating individualized, progressively challenging ball‐based games into elementary school physical education programs and community youth initiatives. To substantiate long‐term efficacy and broader applicability, future large‐scale trials should include extended follow‐up periods and objective PA monitoring.

## Author Contributions

**Neda Mohammad Veysi:** operational execution of the study (investigation), critical final review and editing process (writing – review and editing), ensuring the quality and integrity of the submitted work. **Ayoob Sabaghi:** comprehensive responsibilities across virtually all phases of the research and manuscript development, conceptualization of the study design, development of the methodology, subsequent oversight of its execution (supervision and project administration), resources and data curation, formal analysis, initial software tasks, data validation, initial draft of the manuscript (writing – original draft), subsequent refinement (writing – review and editing). **Behrooz Ebrahimi:** foundational planning through conceptualization, critical oversight in validation and supervision, offering scholarly feedback during the revision phase (writing – review and editing).

## Funding

No funding was received for this manuscript.

## Ethics Statement

All procedures performed in studies involving human participants were in accordance with the ethical standards of the institutional and/or national research committee and with the 1964 Helsinki Declaration and its later amendments or comparable ethical standards.

## Consent

Informed consent was obtained from all individual participants included in the study.

## Conflicts of Interest

The authors declare no conflicts of interest.

## Supporting information


**Supporting Information** Additional supporting information can be found online in the Supporting Information section. Data S1:Representative examples of Heidelberg Ballschule intervention protocols. This supporting information provides representative examples of the game‐based interventions implemented in this study. Due to space limitations, four representative games from the complete set of 15 Heidelberg Ballschule games are described in detail. Both the challenge‐oriented and static groups participated in the same fundamental ball activities; however, the challenge‐oriented group received individually adapted, progressively difficult variations aligned with participants′ optimal challenge points, while the static group performed only the basic versions of each game without progression. The remaining 11 games followed similar progressive structures adapted to the challenge point framework and are available from the corresponding author upon reasonable request.

## Data Availability

The data that support the findings of this study are available from the corresponding author upon reasonable request.
