# Supplementary material for: Differential Effects of Individually Adapted Challenge‐Oriented Heidelberg Ballschule Activities Versus Static Version on Motor Competence and Affective Engagement in 9–11‐Year‐Old Female Children
Source: Biomed Res Int. 2026 May 26;2026:5244454. doi: 10.1155/bmri/5244454 (PMC13202726; doi:10.1155/bmri/5244454)
Supplement: Supplementary file 1 — Supporting Information Additional supporting information can be found online in the Supporting Information section. Data S1:Representative examples of Heidelberg Ballschule intervention protocols. This supporting information provides representative examples of the game‐based interventions implemented in this study. Due to space limitations, four representative games from the complete set of 15 Heidelberg Ballschule games are described in detail. Both the challenge‐oriented and static groups participated in the same fundamental ball activities; however, the challenge‐oriented group received individually adapted, progressively difficult variations aligned with participants′ optimal challenge points, while the static group performed only the basic versions of each game without progression. The remaining 11 games followed similar progressive structures adapted to the challenge point framework and are available from the corresponding author upon reasonable request. [file BMRI-2026-5244454-s001.docx]

**Supplementary Material**

Appendix A: Representative Examples of Heidelberg Ballschule Intervention Protocols

This appendix provides representative examples of the game-based interventions implemented in this study. Due to space limitations, four representative games from the complete set of 15 Heidelberg Ballschule games are described in detail. Both the challenge-oriented and static groups participated in the same fundamental ball activities; however, the challenge-oriented group received individually adapted, progressively difficult variations aligned with participants' optimal challenge points, while the static group performed only the basic versions of each game without progression. The remaining 11 games followed similar progressive structures adapted to the Challenge Point Framework and are available from the corresponding author upon reasonable request.


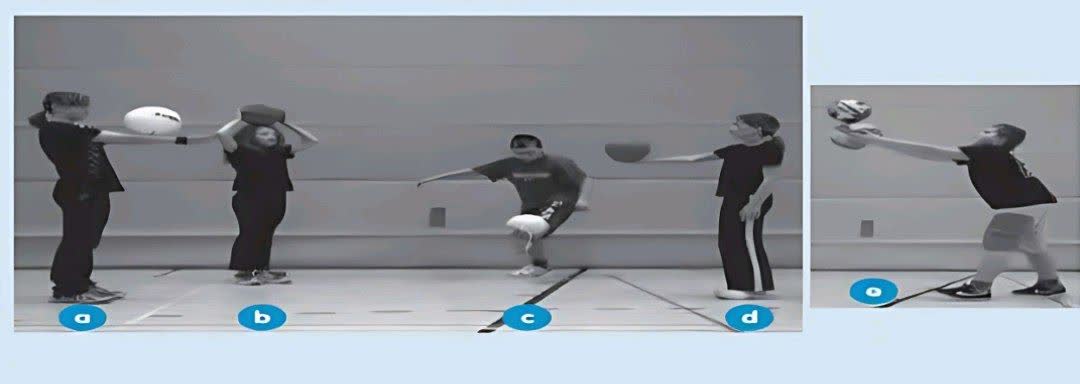


**A1. Ball Balancing Games**

Static Group Version:

- Balancing the ball on the elbow and outstretched hand

- Balancing the ball on the head

- Balancing the ball on the foot

- Balancing the ball on the palm of the hand

Challenge-Oriented Group Version:

Challenge 1: Balancing the ball on the knee, on the arm, or between the shoulders.

Challenge 2: While maintaining the balance of the ball, the subject must sit down and stand up.

Challenge 3: Balancing two balls stacked on top of each other.

Challenge 4: Balancing two balls stacked on top of each other while the subject sits down, stands up, or spins.


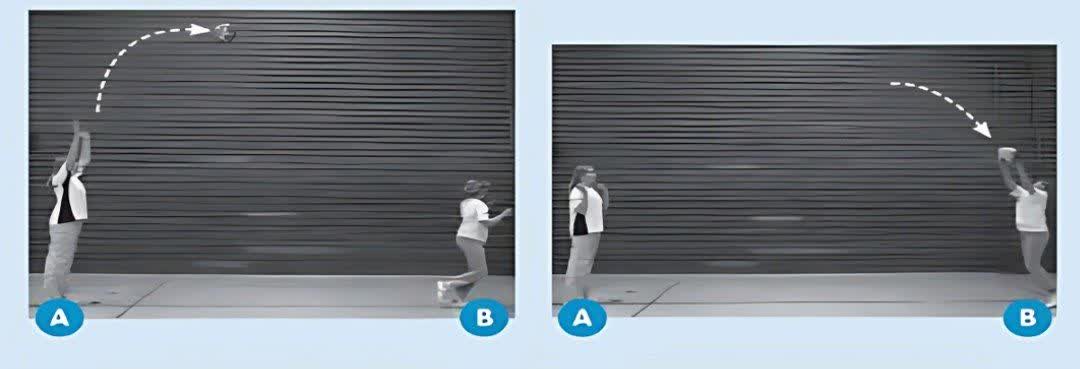


**A2. Throwing and Catching Games**

Static Group Version:

- Two players (A and B) stand several meters apart and throw the ball to each other

- At the same time, additional tasks must be performed (for example, after a relatively low-height throw from A, player B turns and waits to catch the ball mid-flight)

Challenge-Oriented Group Version:

- Before catching the ball, the teammate must show a specific number with their fingers and also announce it aloud

- Before catching, the task specified by A (e.g., stretching the arm = catching in a sitting position, fist = catching in a jumping position, etc.) must be communicated to B

- A throws the ball at a low height, B runs and catches it before it hits the ground, then B repeats the same task

- After throwing, perform a rotation (left-right spin, 360-degree turn, jumping spin)


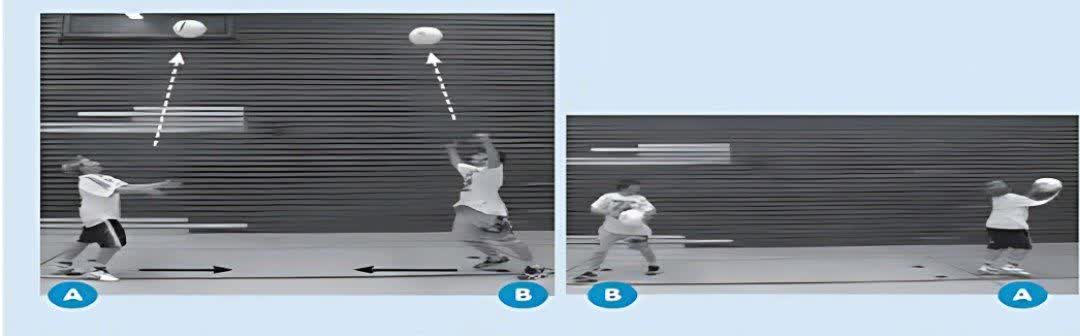


**A3. Simultaneous Exchange Games**

Static Group Version:

- A and B each have a ball and stand facing each other

- At a signal, both players throw their ball vertically into the air, switch places, and each catches the ball thrown by the other

Challenge-Oriented Group Version:

- Perform an additional task while switching places (e.g., touching the teammate's hand, or touching the ground)

- Use a smaller ball

- Change the starting position (e.g., starting from a sitting position)

- Change the catching stance (e.g., rotating to face the teammate or catching while sitting)


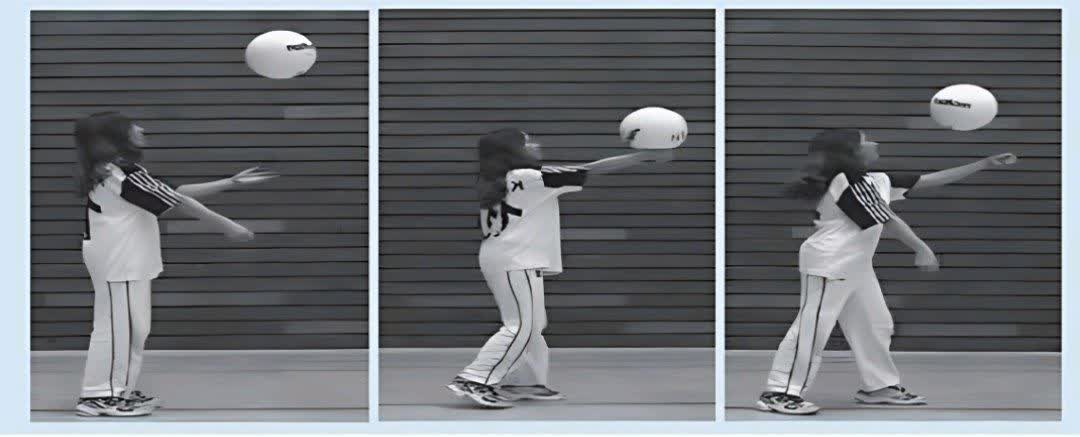


**A4. Keeping the Ball Airborne Games**

Static Group Version:

- The player throws a ball upwards and keeps it in the air by hitting it with the back of their hand

Challenge-Oriented Group Version:

- After throwing the ball, different additional tasks are assigned: squatting, spinning, clapping the soles of the feet together

- Players are asked to use different contact surfaces: upward, downward, back of the hand, fist, palm

- Bouncing the ball at varying heights

- Hitting the ball using the wall
